# Thyroid Stimulating Hormone Triggers Hepatic Mitochondrial Stress through Cyclophilin D Acetylation

**DOI:** 10.1155/2020/1249630

**Published:** 2020-01-06

**Authors:** Xiaolei Wang, Jinbao Mao, Xinli Zhou, Qiu Li, Ling Gao, Jiajun Zhao

**Affiliations:** ^1^Shandong Institute of Endocrine & Metabolic Diseases, Shandong First Medical University & Shandong Academy of Medical Sciences, Jinan 250014, China; ^2^Department of Anesthesiology, Shandong Provincial Hospital Affiliated to Shandong First Medical University, Jinan 250021, China; ^3^Department of Endocrinology, Shandong Provincial Hospital Affiliated to Shandong First Medical University, Jinan 250021, China; ^4^Scientific Center, Shandong Provincial Hospital Affiliated to Shandong First Medical University, Jinan 250021, China; ^5^Shandong Provincial Key Laboratory of Endocrinology and Lipid Metabolism, Jinan 250021, China

## Abstract

**Background & Aims:**

Oxidative stress-related liver diseases were shown to be associated with elevated serum thyroid stimulating hormone (TSH) levels. Mitochondria are the main source of cellular reactive oxygen species. However, the relationship between TSH and hepatic mitochondrial stress/dysfunction and the underlying mechanisms are largely unknown. Here, we focused on exploring the effects and mechanism of TSH on hepatic mitochondrial stress.

**Methods:**

As the function of TSH is mediated through the TSH receptor (TSHR), *Tshr*^−/−^ mice and liver-specific *Tshr* knockout (LKO) mice were used in our study. The thyroid-specific *Tshr* knockout mouse model injected with TSH (TKO+TSH) was used as a mimic for subclinical hypothyroidism (SCH) patients. Hepatic mitochondrial stress and function were analyzed in these mouse models, and the expression of key genes involved in mitochondrial stress was measured.

**Results:**

A relatively lower degree of mitochondrial stress was observed in the livers of *Tshr*^−/−^ mice and LKO mice than those of their littermate counterparts. TSH caused concentration- and time-dependent effects on mitochondrial stress and cyclophilin D (CypD) acetylation in hepatocytes *in vitro*. Microarray and RT-PCR analyses showed that *Tshr*^−/−^ mice had much higher lncRNA-AK044604 expression than their littermate counterparts. The use of the AK044604 overexpression plasmid and SIRT1 agonist proved that TSH aggravates CypD acetylation and mitochondrial stress via lncRNA-AK044604 and SIRT1. An inhibitor of CypD acetylation, cyclosporine A, suppressed TSH-induced hepatic mitochondrial stress and dysfunction.

**Conclusions:**

TSH stimulates hepatic CypD acetylation through the lncRNA-AK044604/SIRT1/SIRT3 signaling pathway, indicating an essential role for TSH in mitochondrial stress in the liver.

## 1. Introduction

An imbalance of reactive oxygen species (ROS) or reactive nitrogen species (RNS) and the antioxidant system results in oxidative stress [1, 2]. Due to the close relationship between oxidative stress and the progression of liver diseases [3–5], antioxidant administration is an increasingly explored therapeutic approach. However, specific therapeutic strategies are lacking because the pathological processes of oxidative stress-related liver diseases have not been fully elucidated.

Subclinical hypothyroidism (SCH) is characterized by elevated TSH and normal free thyroxine (T_4_) levels. In previous studies by our research team and others, serum TSH was proven to be associated with the index of oxidative stress [6, 7]. In addition, we showed the presence of functional TSH receptors (TSHRs) in hepatocytes [8]. We have also done lots of research on TSH and hepatic triglyceride and cholesterol metabolism and devoted ourselves to exploring the mechanisms [9–11]. However, we still do not figure out the effects and mechanisms of TSH on oxidative stress in the liver. In recent years, TSH has been demonstrated to be closely relatedto nonalcoholic fatty liver disease (NAFLD) and liver cirrhosis, two oxidative stress-related liver diseases [12–14], whereas the molecule mechanism remains largely unclear.

Excessive ROS can be produced by mitochondrial respiration, NADPH oxidases, uncoupled nitric oxide synthase, xanthine oxidase, and so on [15]. Hepatocytes are rich in mitochondria, which have an active role in the control of signaling pathways associated with the development of liver diseases, such as NAFLD confirmed by our previous research [16]. Mitochondrial stress or dysfunction contributes to the pathogenesis of liver diseases since it can promote ROS generation, lipid peroxidation, DNA injury, cytokine release, and apoptosis [4]. Furthermore, the vicious cycle of mitochondrial dysfunction and insulin resistance has been shown to play an important role in oxidative stress and metabolic disorders in the liver [5]. Based on the above findings, the mitochondria-related mechanisms may become an important research trend of oxidative stress-related liver diseases. Actually, the complementary relationship between thyroid hormones and mitochondrial biogenesis especially within the frame of cardiac function has been described by many researches [17–19]. Our previous study showed that elevated TSH can trigger mitochondrial perturbations in vascular endothelial cells (ECs), which leads to new and promising methods for targeting ROS elimination to prevent and treat cardiovascular diseases in SCH patients [20]. Although TSH does play a role in mitochondrial stress/dysfunction in ECs [21, 22] and epidermis cells [23], no studies have examined the relationship between TSH and hepatic mitochondrial stress or the underlying mechanism.

The mitochondrial permeability transition pore (mPTP) has a central role in mitochondrial homeostasis; as excessive opening of mPTP will lead to mitochondrial stress, such as impaired mitochondrial respiratory chain function, mitochondrial swelling, and ROS generation [24]. Cyclophilin D (CypD), an intramitochondrial peptidylprolyl-*cis*-*trans*-isomerase, is an initial factor of the mPTP [25]. Increased expression or enhanced activation of CypD will lead to excessive opening of the mPTP. The CypD activity can be modulated by acetylation, which can stimulate excessive mPTP opening [20]. However, CypD deficiency improves mitochondrial function in the mouse cerebral cortex [26], pancreas islets [27], and skeletal muscle [28] and consequently ameliorates cell death and insulin resistance. Recent studies have shown that F1FO ATP synthase oligomycin sensitivity conferring protein (OSCP) is a binding partner of CypD. The interaction of CypD with OSCP modulates F1FO ATP synthase function and mediates mPTP opening. Research suggested that CypD can promote F1FO ATP synthase dysfunction and the resultant mitochondrial deficits in aging brains [25]. In our previous study, we found that CypD activates hepatic mitochondrial stress and triglyceride accumulation, resulting in NAFLD [16], which confirms the role of CypD in oxidative stress-related diseases.

Long noncoding RNA (lncRNA) is a group of nonprotein coding RNAs with the length of over 200 nucleotides. Growing studies have shown that dysregulated lncRNA expression is associated with liver fibrosis [29], failure [30], and carcinoma [31]. Upregulation of the lncRNA metastasis-associated lung adenocarcinoma transcript 1 (MALAT1) is reported to be associated with the promotion of nonalcoholic steatohepatitis- (NASH-) related fibrosis by increasing inflammatory chemokines [32]. MALAT1 may also increase myofibroblast markers by suppressing silent information regulator 1 (SIRT1) expression in liver fibrogenesis [33]. In addition, lncRNA nuclear paraspeckle assembly transcript 1 (NEAT1) can facilitate the expression of the profibrotic gene in liver fibrosis [34]. The study indicated that lncRNAs can not only be used as an early diagnostic indicator of liver failure but also play a regulatory role in an inflammatory response to hepatocyte death and regeneration [30]. LncRNAs play important roles in the biological processes of the occurrence, development, and metastasis of liver cancer. It was proven that upregulated lncRNAs such as URHC [35] and PTTG3P [36] and downregulated lncRNAs PTENP1 [37] and uc002mbe.2 [38] are closely related to hepatocellular carcinoma.

Here, we found that elevated TSH triggers mitochondrial stress in the liver and sheds light on the crucial role of CypD in TSH-induced hepatic mitochondrial perturbations.

## 2. Materials and Methods

### 2.1. Animals

The mice used in our study were approved by the Research Ethics Committee of Shandong Provincial Hospital. Mice were housed in colony cages with a 12 h light/dark cycle in a temperature-controlled environment.

To determine the contribution of TSHR to mitochondrial stress *in vivo*, we utilized the TSHR knockout (*Tshr*^−/−^) mouse model as described in our previous studies [9, 10]. *Tshr*^−/−^ mice and wild-type (littermate counterparts) mice (Jackson Laboratory, USA) were obtained from heterozygous mouse breeding, and the genotype of the mice was determined by PCR. To eliminate the effects of thyroid hormone (TH) deficiency, we supplied exogenous thyroxine (T_4_) to *Tshr*^−/−^ mice after weaning.

Liver-specific *Tshr* knockout (LKO) mice were produced by intercrossing *Tshr^flox/flox^* mice with *Alb-Cre* transgenic mice. This mouse model was generated to check the effect of TSHR in the liver.

To explore the effects of TSH on hepatic mitochondrial stress *in vivo*, we generated a mouse model with elevated TSH. If we directly inject TSH into normal animals, the level of THs (T_4_ and T_3_) will be elevated and serum TSH will be decreased because of the feedback regulation of the hypothalamus-pituitary-thyroid (HPT) axis, which ultimately makes the hormones at normal levels. So, the mice in which TSHR in the thyroid is specifically knocked out were used to produce a mouse model with high TSH. This model, so-called TKO+TSH, is a mimic of subclinical hypothyroidism (SCH). The specific modeling process is as follows. Thyroid-specific *Tshr* knockout (TKO) mice were produced by intercrossing *Tshr^flox/flox^* mice with *TPO-Cre* transgenic mice as described in our previous study [20]. The endogenous THs (T_4_ and T_3_) could not be synthesized in this model, which were supplied with dietary T_4_ after weaning to eliminate the effects of TH deficiency. Detection of serum THs and TSH hormone levels was tested to ensure that they are basically at the normal level [20]. TKO mice were subcutaneously injected with exogenous TSH or solvent for two weeks. This TKO+TSH mouse model can maintain stable elevated TSH levels because TKO inhibits the negative feedback regulation of T_4_ on TSH. With this approach, the elevation of serum TSH level could be controlled by TSH injection without altering serum TH levels, so the effects of TSH *in vivo* could be observed.

TKO mice received successive intraperitoneal (ip.) injection of cyclosporine A (CsA, Novartis, 15 mg·kg^−1^·d^−1^) or PBS for another 6 weeks, and TSH was given through subcutaneous (sc.) injection from the 12^th^ to the 14^th^ week to generate TKO+TSH+CsA mice.

### 2.2. Isolation of Mitochondria

Mitochondria were isolated from the livers with a previously described protocol with modifications [26]. For the mitochondrial swelling, MDA, or western blot analyses, mitochondria were isolated by centrifuging liver cells at 1300 g for 5 minutes at 4°C. We adjusted the supernatant to 15% Percoll and recentrifuged it at 36500 g for 20 minutes, 4°C (Hitachi, Japan) [16]. The supernatant was decanted, and the pellet was resuspended in mitochondrial isolation buffer and centrifuged at 10000 g for 10 minutes at 4°C. Mitochondrial protein concentration was determined at 540 nm by NanoDrop 2000c (Thermo Scientific).

Other methods are listed in Supplementary Materials ([Supplementary-material supplementary-material-1]).

### 2.3. Statistical Analyses

The analyses were performed using SPSS 19.0 software (Chicago, IL, USA). The results are reported as the mean ± SD. The comparison of different groups was performed using one-way ANOVA. Two-tailed *p* < 0.05 was considered significant.

## 3. Results

### 3.1. TSH Induces Mitochondrial Stress *In Vivo* and *In Vitro*

**Figure 1:**
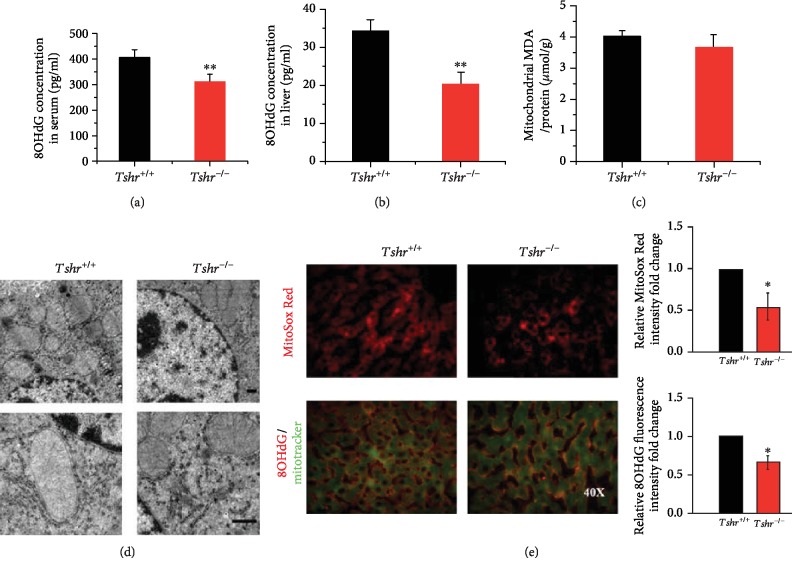
Hepatic mitochondrial stress in *Tshr^−/−^* and *Tshr^+/+^* mice. *Tshr^−/−^* mice and *Tshr^+/+^* (littermate counterparts) mice were fed a normal diet and were analyzed for mitochondrial stress. (a, b) ELISAs of 8OHdG levels in serum and liver. (c) Mitochondrial MDA levels were measured using the TBA method, and the concentrations of MDA are expressed as *μ*mol/g protein. (d) Representative images of TEM (the upper is 7500x and the lower is 20000x). (e) Representative images of MitoSox Red staining (upper), and double-staining (lower) showed 8OHdG (red) and MitoTracker (green) in the mouse livers by immunofluorescence microscopy. The data are presented as the mean ± SD. ^∗^*p* < 0.05, ^∗∗^*p* < 0.01 versus *Tshr^+/+^* mice.

The function of TSH is mediated through the highly specific TSHR [39]. In our previous study, we demonstrated the presence of functional TSHRs in hepatocytes [8]. To determine the effect of TSH on mitochondrial stress, we generated a *Tshr* knockout (*Tshr^−/−^*) mouse model, which is resistant to TSH. This model is characterized by severe hypothyroidism because it cannot synthesize THs. To exclude the potential influence of TH deficiency, mice were supplemented with T_4_ powder [40]. *Tshr^−/−^* mice supplemented with T_4_ have normal serum levels of T_4_ and TSH, which was demonstrated in our study [10]. *Tshr^−/−^* mice exhibited less oxidative stress damage than *Tshr^+/+^* (littermate counterparts) mice, as evidenced by decreased 8-hydroxy-deoxyguanosine (8OHdG) in the serum and liver (Figures 1(a) and 1(b)), which indicates DNA oxidation damage at the overall level of cells. The results showed that oxidative injury was decreased in *Tshr^−/−^* mice.

As mitochondria are the main source of ROS, we examined mitochondrial stress in the liver. When mitochondrial lipid peroxidation damage was measured, we failed to observe differences in hepatic mitochondrial malondialdehyde (MDA) levels in the two mouse groups (Figure 1(c)). Transmission electron microscopy (TEM) analysis showed that mitochondrial swelling and vacuolation were attenuated in the livers of *Tshr*^*-/*-^ mice to a certain degree (Figure 1(d)), although not so obvious. Given that, we used another mouse model with high TSH levels *in vivo* (TKO+TSH mouse model) to check hepatic mitochondrial abnormalities. To evaluate mitochondrial ROS generation, we used MitoSox Red, a unique fluorogenic dye that allows for selective detection of superoxide production in the mitochondria. *Tshr^−/−^* mice showed less MitoSox staining than *Tshr^+/+^* mice (Figure 1(e), upper), indicating that the absence of TSHR reduced mitochondrial ROS generation. The mitochondrial 8OHdG was also decreased in *Tshr^−/−^* mice (Figure 1(e), lower). In summary, these findings suggest a relatively low level of mitochondrial stress in *Tshr^−/−^* mice.

Next, we intended to verify the effect of TSH *in vitro*. HepG_2_ cells are derived from hepatocytes and retain many of the properties of hepatocytes [41]. This human cell line has a wide variety of liver-specific metabolic responses to different types of stimuli [9] and could effectively mimic the *in vivo* environment for metabolism [42]. Therefore, the HepG_2_ cell line was selected to perform *in vitro* experiments. TSH caused a dose-dependent effect on intracellular mitochondrial ROS generation in HepG_2_ cells ([Supplementary-material supplementary-material-1]). Therefore, we chose 4 *μ*M TSH for further experiments. Accordingly, TEM analysis revealed a certain degree of structural damage of mitochondria in TSH-treated cells ([Supplementary-material supplementary-material-1]). However, the effect of TSH on ROS generation linking TSH with mitochondrial function remains unknown. The loss of mitochondrial membrane potential and the dysfunction of the mitochondrial respiratory chain can cause mitochondrial disorders [24], resulting in excessive mitochondrial ROS generation. TSH aggravated mitochondrial dysfunction as evidenced by JC-1 fluorescence staining ([Supplementary-material supplementary-material-1]) and the Mito Stress Test ([Supplementary-material supplementary-material-1]), which showed that TSH decreased the oxygen consumption ratios (OCRs) of the maximal respiration and spare respiratory capacity without changing the OCR of ATP production. These results confirmed that TSH directly increased mitochondrial stress *in vitro* and TSH-mediated mitochondrial stress is related to the respiratory chain dysfunction.

### 3.2. LncRNA/SIRT1/SIRT3 Is Involved in TSH-Mediated Hepatic Mitochondrial Stress

**Figure 2:**
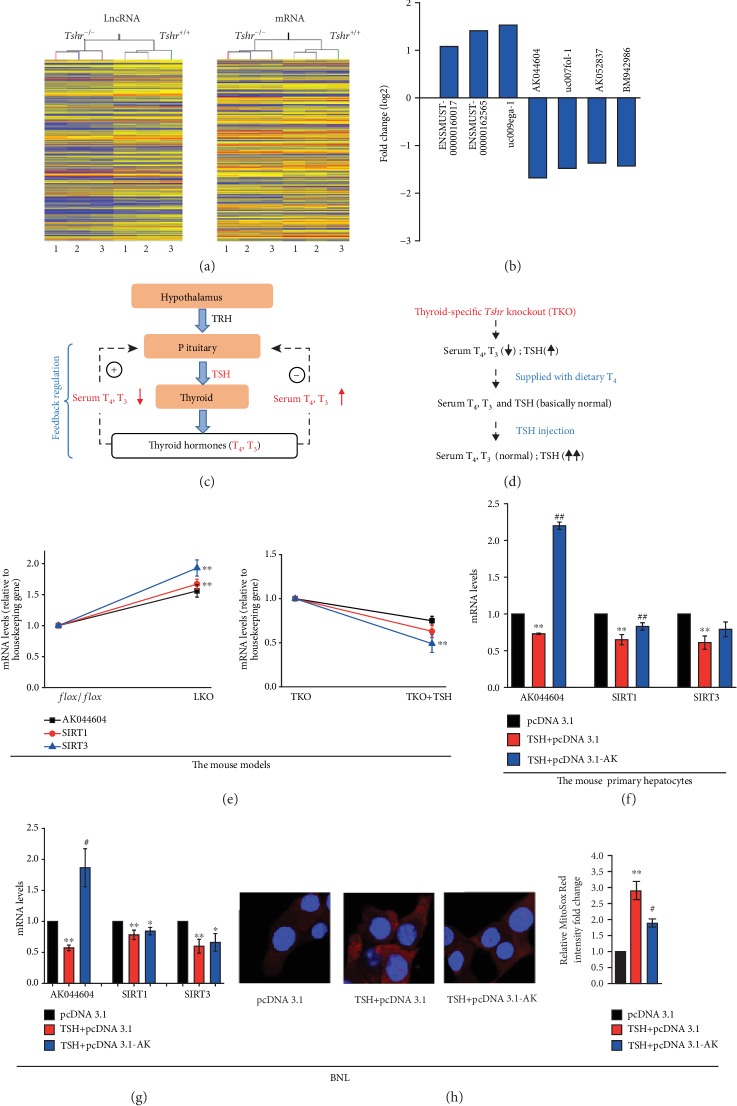
Effect of lncRNA *in vivo* (a, b, e) and *in vitro* (f–h). (a) Heat map and unsupervised hierarchical clustering of lncRNAs. (b) Fold change of seven lncRNAs selected from differentially expressed lncRNAs in the microarray. (c) Schematic illustration of the regulation of the hypothalamus-pituitary-thyroid (HPT) axis. The paraventricular nucleus in the hypothalamus releases TRH, which acts on the pituitary to stimulate TSH synthesis. TSH acts on thyrocytes to stimulate the thyroid hormones T_4_ and T_3_, which act on the pituitary to inhibit TSH synthesis and release, and this feedback regulation is the main regulatory mechanism of thyroid function. + represents stimulation, and - represents inhibition. (d) Schematic illustration of TKO+TSH mouse model building. (e) The mRNA expression of lncRNA-AK044604, SIRT1, and SIRT3 in the livers of liver-specific *Tshr* knockout (LKO) mice and thyroid-specific *Tshr* knockout mice injected with TSH (TKO+TSH). ^∗∗^*p* < 0.01 vs. the flox or TKO group. All mice in the TKO group were supplied with dietary T_4_ to ensure serum THs, and TSH hormones are basically at the normal level. The mRNA expression in normal mouse primary hepatocytes (f) and BNL (g) induced by TSH or AK044604 overexpression plasmid (pcDNA3.1-AK). (h) Representative images of MitoSox Red staining in BNL cells by confocal immunofluorescence microscopy (40x). The data are presented as the mean ± SD. ^∗^*p* < 0.05, ^∗∗^*p* < 0.01 vs. the control group; ^#^*p* < 0.05, ^##^*p* < 0.01 vs. the TSH+pcDNA3.1 group.

Many lncRNAs have been found to be aberrantly expressed in oxidative stress-related liver diseases, including hepatocellular carcinoma [43] and liver fibrosis [44]. To explore the mechanism of TSH-induced mitochondrial stress, we chose 3 matching pairs of *Tshr^+/+^* and *Tshr*^−/−^ mouse liver tissues for microarray analysis of lncRNAs and mRNAs. We set a threshold as a fold change that is larger than 1.5 and a *p* value that is smaller than 0.05 and found that there were 16043 dysregulated lncRNAs and 13146 dysregulated mRNAs in the livers of *Tshr^+/+^* and *Tshr^−/−^* mice (Figure 2(a)), indicating that the lncRNA and mRNA levels were significantly different between the two groups. To validate the microarray analysis findings, we selected the top 7 lncRNAs from the differentially expressed lncRNAs, and their expression levels were detected by real time- (RT-) PCR (data not shown). The PCR data confirmed the microarray results (Figure 2(b)). Among the lncRNAs differentially expressed in the tissues, one lncRNA, accession number AK044604 in NCBI, was significantly upregulated in *Tshr^−/−^* mice. Studies have indicated that lncRNAs play regulatory roles mainly by binding to their target gene products [45]. For example, AK044604 in our study was estimated to regulate SIRT1 by microarray analysis. The microarray analysis showed that the relationship between AK044604 and the promoter region of SIRT1 is “bidirectional” (data not shown). NCBI's BLAST alignment showed that a segment of 497 nucleotides was found 100% identical in AK044604 and the promoter region of SIRT1 ([Supplementary-material supplementary-material-1]). The result indicated that AK044604 might mediate SIRT1 mRNA expression via binding to this site, which needs further confirmation. The SIRT1/SIRT3 pathway can regulate mPTP opening and mitochondrial function in airway smooth muscle cells [46]. Furthermore, the inactivation of CypD induced by SIRT3, a major deacetylase that regulates mitochondrial metabolism, is necessary for mitochondrial function [20].

Next, we validated the mRNA expression in LKO and TKO+TSH mouse models. In Materials and Methods, we described the HPT axis under physiological conditions (Figure 2(c)) and the TKO+TSH mouse model in detail (Figure 2(d)). We detected key gene expression in the two mouse models by RT-PCR (Figure 2(e)). To eliminate the effects of other tissues, we used LKO mice to validate the microarray analysis results. The mRNA levels of AK044604, SIRT1, and SIRT3 were increased in LKO mice compared to *flox/flox* (littermate counterparts) mice. To determine the direct effects of TSH on key gene expression, we established a mouse model with high TSH levels *in vivo* to inhibit the negative feedback regulation of T_4_ on TSH. Therefore, TKO mice were injected with TSH to maintain stable elevated TSH levels. The mRNA expression levels of AK044604, SIRT1, and SIRT3 were decreased in TKO+TSH mice compared to TKO mice. Furthermore, we examined the effect of TSH on mRNA expression of AK044604 and SIRTs in normal mouse primary hepatocytes to check whether it was consistent with results *in vivo*. To eliminate the limitations of primary hepatocytes and to ensure the expansibility of the results, we also used mouse embryonic liver cells (BNL) for RT-PCR analysis. Overexpression of AK044604 stabilized mRNA expression of SIRT1 and SIRT3 in mouse primary hepatocytes (Figure 2(f)) and BNL cells (Figure 2(g)) and suppressed mitochondrial ROS generation *in vitro* exposed to TSH (Figure 2(h)), which indicated that the expression of lncRNA-AK044604 and SIRT1/3 might mediate TSH-induced mitochondrial stress.

### 3.3. TSH Increases CypD Acetylation through SIRT1/SIRT3 Signaling

**Figure 3:**
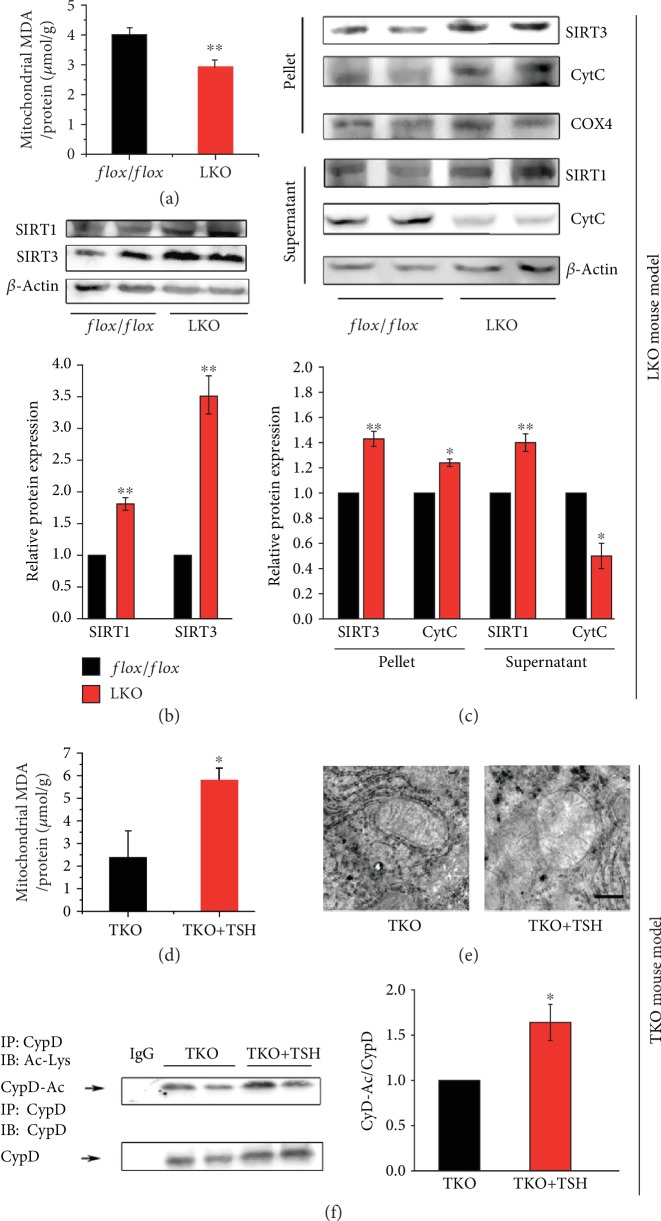
TSH stimulates mitochondrial stress via the SIRT1/SIRT3/CypD signaling pathway. (a, d) Mitochondrial MDA levels in the liver. (b) Representative images of immunoblotting (upper) and quantification (lower) for SIRT1 and SIRT3 in total liver protein. (c) Representative images of immunoblotting (upper) and quantification (lower) for cytochrome c (CytC) and SIRT1/3 in the mitochondria (pellet) and cytosolic (supernatant) fractions. (e) Representative images of TEM (20000x). (f) Representative images of immunoprecipitation (left) and quantification (right) for CypD acetylation. All mice in the TKO group were supplied with dietary T_4_ to ensure that serum THs and TSH hormone are basically at the normal level. The data are presented as the mean ± SD. ^∗^*p* < 0.05, ^∗∗^*p* < 0.01.

SIRT3 can deacetylate CypD [20], and CypD acetylation-mediated mPTP opening can aggravate mitochondrial stress. To confirm the effect of TSH, we analyzed hepatic mitochondrial stress and SIRT1/SIRT3 expression. The MDA levels (Figure 3(a)) and cytochrome C (CytC) release from the mitochondria (pellet) to the cytoplasm (supernatant) (Figure 3(c)) were reduced in LKO mice compared to *flox/flox* mice. In addition, SIRT1 and SIRT3 protein expression was increased in LKO mice, regardless of total proteins or mitochondrial/cytoplasmic proteins (Figures 3(b) and 3(c)). There are many other proteins (besides CypD) whose activation plays an important role in mitochondrial respiratory function, for example, the oxidative phosphorylation system (OXPHOS), which consists of five multiheteromeric complexes embedded in the inner mitochondrial membrane. The first four complexes including CytC oxidase (COX), together with CytC, are the main components of the mitochondrial respiratory chain. Assembly factors of OXPHOS have been reported in the literature as responsible for many mitochondrial diseases in humans [47]. We used COX IV as an internal control of mitochondrial protein and tested the release of CytC to check the extent of respiratory chain injury (Figure 3(c)). However, that was insufficient, and further studies are needed to investigate whether the activation of OXPHOS proteins or respiratory complexes are associated with TSH-induced mitochondrial stress in the liver. Furthermore, the TKO+TSH mice were used to determine the direct effect of TSH on mitochondrial stress and key gene expression *in vivo*. The MDA levels (Figure 3(d)) were increased in TKO+TSH mice, along with swollen mitochondria and destroyed mitochondrial membrane (Figure 3(e)) and stimulated CypD acetylation (Figure 3(f)), compared to those of TKO mice.

The mouse primary hepatocytes and HepG_2_ cells were used to test the mechanism *in vitro*. We found that TSH stimulated CytC release and CypD acetylation and downregulated SIRT1 and SIRT3 protein expression ([Supplementary-material supplementary-material-1] and [Supplementary-material supplementary-material-1]). The SIRT1 agonist SRT1720 suppressed CypD acetylation ([Supplementary-material supplementary-material-1]) and decreased mitochondrial MDA levels ([Supplementary-material supplementary-material-1]) in TSH-treated cells. These results suggested that TSH can induce CypD acetylation through SIRT1/SIRT3 signaling.

### 3.4. Therapeutic Effect of CypD Acetylation Inhibitor in Hepatic Mitochondrial Stress

Our results above showed that TSH-induced mitochondrial stress is characterized by DNA oxidation and mitochondrial ROS generation. Next, we checked where increased mitochondrial ROS came from.

**Figure 4:**
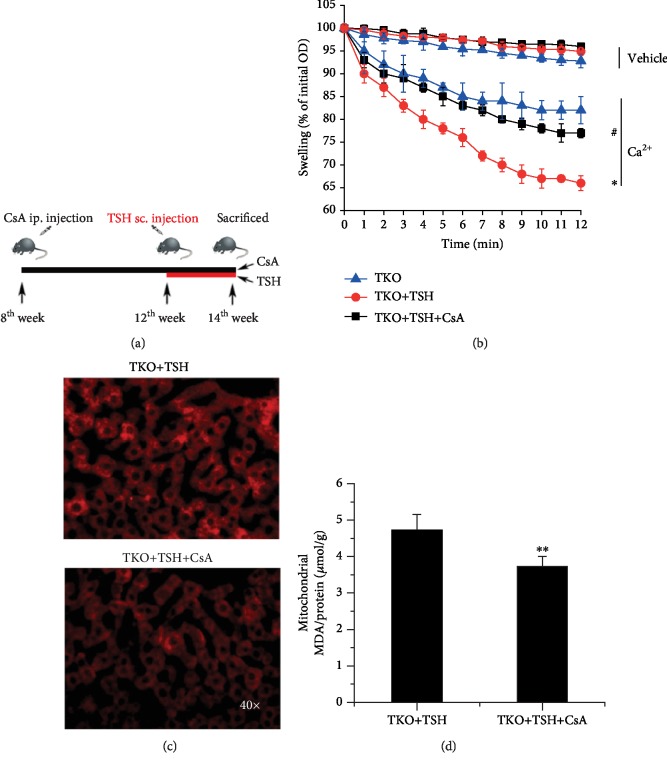
The suppressive effect of a CypD acetylation inhibitor on hepatic mitochondrial stress. (a) Eight-week-old TKO mice received successive intraperitoneal (ip.) injection of CsA for another 6 weeks, and TSH was given through subcutaneous (sc.) injection at the 12^th^ week. (b) Mitochondrial swelling analysis. ^∗^*p* < 0.05 versus TKO mice, ^#^*p* < 0.05 versus TKO+TSH mice. (c) Representative images of MitoSox Red staining in the mouse livers by immunofluorescence microscopy. (d) Mitochondrial MDA levels. ^∗∗^*p* < 0.01 versus TKO+TSH mice. All mice in the TKO group were supplied with dietary T_4_ to ensure serum THs and TSH hormone are basically at the normal level. The data are presented as the mean ± SD.

CsA is emerging as the most notable inhibitor of mPTP opening by binding to a special domain of CypD, which is adjacent to the acetylation site [48]. We used CsA (Sandimmune) to determine whether CypD plays an important role in TSH-induced hepatic mitochondrial stress. Eight-week-old TKO mice received successive ip. injection of CsA for another 6 weeks, and TSH was given through sc. injection at the 12^th^ week (Figure 4(a)). Mitochondrial swelling reflects excessive mPTP opening, which results in mitochondrial ROS generation, DNA injury, cytokine release, and apoptosis [3]. Hepatic mitochondria swelling of TKO+TSH mice is more severe than that of TKO mice. The mitochondria of CsA-injected mice were more resistant to swelling and permeability transition in response to Ca^2+^ than those of TKO+TSH mice. Injection of CsA suppressed mitochondrial swelling (Figure 4(b)), ROS generation (Figure 4(c)), and MDA levels (Figure 4(d)) in the livers of the TKO+TSH mice. Here, we confirmed that TSH-mediated mitochondrial ROS generation is at least partly from the mitochondrial respiratory chain, thus suggesting that CypD will be an important therapeutic target for TSH-induced hepatic mitochondrial stress.

## 4. Discussion

Proper mitochondrial function is critical for the maintenance of cell life because the mitochondrion is the energy factory of the cell. Here, we found that TSH can induce hepatic mitochondrial stress *in vivo* and *in vitro* via the lncRNA-AK044604/SIRT1/SIRT3/CypD signaling pathway. Our findings indicated the mitochondrial stress inducer role of TSH and the potential therapeutic effect of CypD inhibitors in oxidative stress-related liver disease.

Oxidative stress has been reported to be the collective pathophysiological mechanism of many liver diseases. Our previous research proved that CypD stimulates mPTP excessive opening, subsequently causing mitochondrial oxidative stress and results in enhanced hepatic steatosis, which links oxidative stress with liver diseases [16]. Previous studies have also demonstrated that TSH is associated with oxidative stress at the whole body level or in specific tissues [6, 7, 12–14]. To determine the degree of oxidative stress *in vivo*, we used *Tshr*^−/−^ mice, which were supplied with dietary thyroid extract (T_4_) after weaning as described previously to exclude the potential role of THs [49]. Excessive production of ROS exceeds the cellular antioxidant capacity and results in nucleic acid and lipid damage, represented by 8OHdG and MDA levels, respectively. We found that 8OHdG in serum and liver was decreased in *Tshr*^−/−^ mice compared to *Tshr*^+/+^ mice, while MDA levels were reduced in LKO mice but not in *Tshr*^−/−^ mice, indicating that oxidative stress was ameliorated in *Tshr*^−/−^ mice and LKO mice. Mitochondrial stress was also tested as the main source of ROS is mitochondria. The decrease in mitochondrial ROS generation and the improvement of mitochondrial swelling indicated an ameliorative mPTP function in *Tshr*^−/−^ and LKO mice. These results suggested the crucial role of the TSH receptor (TSHR) in TSH-induced mitochondrial stress, especially hepatic TSHR. In addition, considering the complexity of the *in vivo* conditions, we used mouse primary hepatocytes and HepG_2_ cells to observe the direct effects of TSH. TSH induced the destruction of the mitochondrial structure and the loss of the mitochondrial membrane potential, resulting in mitochondrial ROS generation and mitochondrial stress damage, which indicates that TSH can directly trigger hepatic mitochondrial stress.

LncRNAs are a group of noncoding RNA transcripts that are reported to be associated with many oxidative stress-related liver diseases [45, 46, 49, 50]. Although they have little or no protein-encoding function normally, lncRNAs are considered to play a potential role in various diseases, such as metabolic disorders [51], cardiac diseases [52], and tumors [53], via the regulatory function. However, whether TSH could promote hepatic mitochondrial stress through lncRNA regulation has not been previously elucidated. In the present study, we found that *Tshr*^−/−^ mice had much higher lncRNA-AK044604 expression than their littermate counterparts, and AK044604 in our study was shown to regulate SIRT1 by microarray analysis, which was validated by RT-PCR.

Studies showed that forkhead box protein O1 (FoxO1) binding to the SIRT1 promoter region can be increased in ischemia-reperfusion. Resveratrol can restore SIRT1 activity and NAD^+^ level by an adenosine monophosphate-activated protein kinase- (AMPK-) dependent mechanism [54]. AMPK is a known positive modulator of sirtuin activity [55], and our previous study proved that TSH can inhibit AMPK activity in the liver [56]. In this study, we conformed the relationship between TSH and sirtuin and the regulatory role of TSH on lncRNA-AK044604, thus implying that TSH may act on AK044604 through AMPK. However, the specific mechanism and binding site still need further study.

Sirtuin is a member of NAD-dependent protein deacetylases that is implicated in many cellular physiological functions, and the most studied sirtuins are SIRT1 (nuclear) and SIRT3 (mitochondrial). Researchers have suggested that SIRT1 can regulate mitochondrial bioenergetics through the induction of SIRT3 expression [57], which emphasizes the synergistic effect between nuclear SIRT1 and mitochondrial SIRT3. Similar to this, we found that SIRT1 and SIRT3 mRNA levels were increased in *Tshr^−/−^* mice and LKO mice compared to their littermate counterparts.

To mimic the human disease states of high TSH, we generated a mouse model in which TSHR in the thyroid is specifically knocked out (TKO) to ensure that endogenous THs could not be synthesized. THs have been reported to influence metabolism [58], and changes of THs may have adverse effects on liver metabolism [44]. To eliminate the effects of TH deficiency, we supplied exogenous T_4_ to TKO mice to maintain normal serum TH levels. The TKO mouse model injected exogenous TSH can maintain stable elevated TSH levels because TKO inhibits the negative feedback regulation of T_4_ on TSH. With this approach, the elevation of the serum TSH level could be controlled by injection of TSH without altering serum TH levels, so the effects of TSH *in vivo* could be observed. Our results showed that AK044604, SIRT1, and SIRT3 mRNA expression was decreased in TKO+TSH mice compared to TKO mice, while overexpression of AK044604 stabilized the mRNA expression of SIRT1 and SIRT3 and suppressed the mitochondrial ROS generation in TSH-induced hepatocytes. This indicated that TSH can aggravate mitochondrial ROS accumulation via downregulating AK044604 and SIRT, although this should be verified *in vivo*.

SIRT1/3 protein levels and deacetylase activities were demonstrated to be decreased in aortic smooth muscle cells following severe shock along with aggravated mitochondrial damage [46]. Consistent with this study, we found that mitochondrial stress was suppressed, while SIRT1 deacetylase activity and SIRT1/3 protein expression were increased in LKO mice compared to *flox/flox* mice, implying that TSH-induced hepatic mitochondrial stress is mediated by decreased SIRT1 expression and activation, which confirms our microarray analysis results. The results above showed that TSH downregulates SIRT1/3 expression through lncRNA-AK044604, resulting in increased hepatic mitochondrial ROS accumulation, which confirms the regulatory role of lncRNA/SIRT1/SIRT3 in hepatic mitochondrial stress.

Acetylation is an important posttranslational modification of CypD, which modulates the translocation of CypD from the matrix to the inner membrane to facilitate the mPTP opening [46, 59]. In our study, the increment in CypD acetylation under TSH exposure demonstrated that elevated TSH stimulated mPTP opening mainly by enhancing CypD activity. The inactivation of CypD induced by SIRT3 is necessary for mitochondrial function maintenance [60]. Actually, the mitochondrial function is critical for setting the NADH/NAD+ balance, because the sirtuin deacetylases consume NAD+ as a cosubstrate. NADH/NAD+ ratio can mediate the function of F-ATP synthase subunit OSCP. Actually, SIRT3 can directly modulate the mPTP by acetylation of OSCP besides the deacetylation of CypD [61]. So, the underlying mechanisms of SIRT3 in mPTP opening and mitochondrial function need more explanation. Our findings showed that hepatic mitochondrial functions, SIRT1 deacetylase activity, and CypD deacetylation were downregulated in TKO+TSH mice compared to TKO mice. Furthermore, TSH upregulated mitochondrial stress and CypD acetylation *in vitro*, while the SIRT1 agonist SRT1720 suppressed these effects. Our results indicated that SIRT1/SIRT3 can mediate TSH-induced CypD acetylation and mitochondrial stress *in vivo* and *in vitro*, which is consistent with references [20, 60]. The present results provided evidence that TSH, through TSHR, increased hepatic CypD acetylation via the lncRNA-AK044604/SIRT1/SIRT3 pathway, leading to hepatic mitochondrial stress.

CsA is an inhibitor of CypD activation, whose binding pocket is adjacent to the acetylation site of CypD [62]. Researchers has identified that CsA can block mPTP formation and partially protect against myocardial infarction and atherosclerotic plaque formation [63, 64]. In our previous study, we found that pharmacological inhibition of CypD by CsA not only attenuated HFD-induced mitochondrial ROS generation but also ameliorated hepatic steatosis [16]. These results suggest that CsA can be, to a certain degree, a potential therapeutic tool for hepatic mitochondrial stress. Considering the wide pharmacological effects of CsA as an immunosuppressant [65], it is necessary to eliminate the possibility that decreased hepatic mitochondrial stress was derived from secondary effects. The specific inhibition of CypD *in vitro* should be used to see whether there will be consistent results. The results suggested that CypD could be a therapeutic target for TSH-induced hepatic mitochondrial stress.

## 5. Conclusions

**Figure 5:**
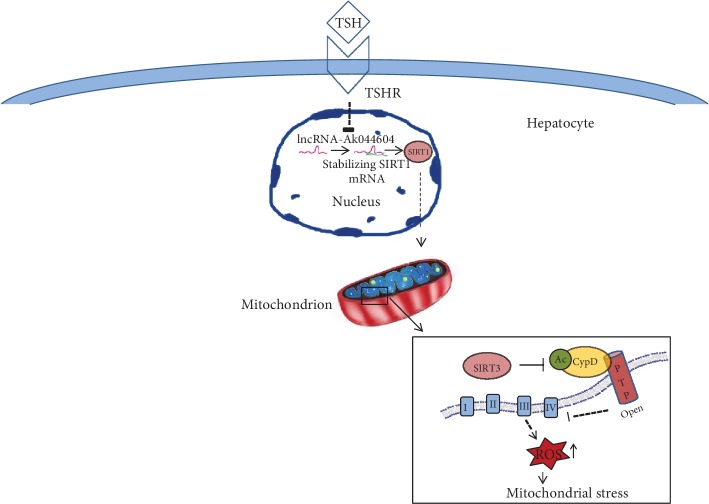
Schematic illustration of the mechanism underlying TSH-induced mitochondrial stress. LncRNA-AK044604 downregulated by TSH can stabilize SIRT1 expression, while the SIRT1/SIRT3 pathway regulates mitochondrial function via deacetylation of CypD in hepatocytes. TSH induced hepatic CypD acetylation through the lncRNA-AK044604/SIRT1/SIRT3 signaling pathway.

In conclusion, we propose that TSH downregulates lncRNA-AK044604 expression and SIRT1/SIRT3 deacetylase activity, subsequently causing increased CypD acetylation and hepatic mitochondrial stress (Figure 5). These results identified the effect of TSH and suggested that CypD might be a novel target in oxidative stress-related liver disease, with pathological implications for the pathogenesis of abnormal oxidative stress in SCH patients.

## Data Availability

The data used to support our findings in this study are available from the corresponding author upon request.

## Supplementary Materials

Supplementary Materials Figure S1: TSH triggers mitochondrial stress in HepG_2_ cells. HepG_2_ cells were cultured in pretreatment medium with or without TSH. (a) Representative images of MitoSox Red staining in HepG_2_ cells by immunofluorescence microscopy. H_2_O_2_ was used as a positive control. (b) Representative images of TEM showing impairment of mitochondria in TSH-treated cells. (c) JC-1 staining. (d) Cells were seeded in XF Cell Culture Microplates. Mito Stress Test parameters from Wave data were exported to Excel, and ATP production, the maximal respiration, and spare respiratory capacity were analyzed. The data are presented as the mean ± SD. ^∗^*p* < 0.05, ^∗∗^*p* < 0.01 versus the control group. Figure S2: NCBI's BLAST alignment showed that a segment of 497 nucleotides was found 100% identical in AK044604 and the promoter region of SIRT1. Figure S3: SIRT1/SIRT3/CypD signaling is involved in TSH-induced mitochondrial stress in vitro. (a) Representative images of immunoblotting (upper) and quantification (lower) in the mitochondrial (pellet) and cytosolic (supernatant) fractions of TSH-induced mouse primary hepatocytes. (b–d) HepG_2_ cells. (b, c) Representative images of immunoblotting (upper) and quantification (lower) for CypD acetylation. (d) MDA levels. The data are presented as the mean ± SD. ^∗^*p* < 0.05, ^∗∗^*p* < 0.01 versus the control group; ^#^*p* < 0.05, ^##^*p* < 0.01 vs. the TSH group. Table S1: the oligonucleotide primer list.
