# Supplementary material for: Thyroid Stimulating Hormone Triggers Hepatic Mitochondrial Stress through Cyclophilin D Acetylation
Source: Oxid Med Cell Longev. 2020 Jan 6;2020:1249630. doi: 10.1155/2020/1249630 (PMC6970002; doi:10.1155/2020/1249630)
Supplement: Supplementary Materials — Figure S1: TSH triggers mitochondrial stress in HepG2 cells. HepG2 cells were cultured in pretreatment medium with or without TSH. (a) Representative images of MitoSox Red staining in HepG2 cells by immunofluorescence microscopy. H2O2 was used as a positive control. (b) Representative images of TEM showing impairment of mitochondria in TSH-treated cells. (c) JC-1 staining. (d) Cells were seeded in XF Cell Culture Microplates. Mito Stress Test parameters from Wave data were exported to Excel, and ATP production, the maximal respiration, and spare respiratory capacity were analyzed. The data are presented as the mean ± SD. ∗p < 0.05, ∗∗p < 0.01 versus the control group. Figure S2: NCBI's BLAST alignment showed that a segment of 497 nucleotides was found 100% identical in AK044604 and the promoter region of SIRT1. Figure S3: SIRT1/SIRT3/CypD signaling is involved in TSH-induced mitochondrial stress in vitro. (a) Representative images of immunoblotting (upper) and quantification (lower) in the mitochondrial (pellet) and cytosolic (supernatant) fractions of TSH-induced mouse primary hepatocytes. (b–d) HepG2 cells. (b, c) Representative images of immunoblotting (upper) and quantification (lower) for CypD acetylation. (d) MDA levels. The data are presented as the mean ± SD. ∗p < 0.05, ∗∗p < 0.01 versus the control group; #p < 0.05, ##p < 0.01 vs. the TSH group. Table S1: the oligonucleotide primer list. [file 1249630.f1.docx]

**Supplementary Materials and Methods**

***Mitochondrial Swelling***

A calcium-triggered mitochondrial swelling assay was performed according to a previous method [1] with modifications. 20 μg freshly isolated liver mitochondria were suspended in 200 μl swelling assay buffer (mmol/L: KCl 150, HEPES 5, K2HPO4 2, glutamate 5, malate 5, pH 7.2), and the swelling was stimulated by calcium (1 μmol•mg^-1^ protein) at 540 nm on a microplate reader SpectraMax M2/M2e (Molecular Devices, LLC).

***Mito-Stress Test***

An XF Cell Culture Microplate (Seahorse Bioscience) was used in our study. The oxygen consumption ratios (OCRs) of ATP production, the maximal respiration, and spare respiratory capacity were analyzed. All procedures are followed Manufacturer's instructions. We measured OCR with XF96 Analyzer (Seahorse Bioscience). HepG_2_ cells were seeded overnight at 8000 cells per well on Seahorse XF96 cell culture microplate (Seahorse Bioscience). After treated, the cells was incubated in low buffered non-bicarbonated assay medium (XF base medium with 2 mmol/L Glutamine, 1 mmol/L Sodium Pyruvate and 25 mmol/L Glucose) for 1 h in a non-CO2 incubator at 37 °C before measuring. OCR was measured for 3 periods with a mixing of 3 min in each cycle.

***MDA***

The mitochondrial MDA levels were measured using a commercial kit (Beyotime, Shanghai, China) according to the manufacturer's directions. Mitochondria were isolated from the livers or cells as described before. The supernatant was reacted with thiobarbituric acid (TBA) and the reaction product was spectrophotometrically measured at 535 nm (SpectraMax M2/M2e, Molecular Devices, LLC). The concentrations of MDA were normalized to the corresponding total protein.

***JC-1 Staining***

The cells were treated and stained using a commercial kit (Beyotime, Shanghai, China). Red fluorescence is from JC-1 aggregates in healthy mitochondria with polarized inner mitochondrial membranes, while green fluorescence is emitted by cytosolic JC-1 monomers and indicates membrane potential dissipation. Merged images indicate the colocalization of JC-1 aggregates and monomers.

***Measurement of 8OHdG***

Tissue blocks were cut into sections, and cryostat sections were doubly stained with MitoTracker (Invitrogen, 1:2000) and 8OHdG (Abcam, 1:50). The sections were examined under a fluorescence microscope after mounting.

In addition, the 8OHdG Concentration levels in the liver and serum were measured using a commercial kit (Cusabio, China) according to the manufacturer's directions.

***Transmission Electron Microscopic Analysis***

The pretreatment of liver tissues and the cells was described as in our previous studies [2, 3]. Briefly, liver tissues were fixed for 2 h with 2.5% glutaraldehyde in 0.1 M cacodylate buffer, washed three times with 0.1 M cacodylate buffer and fixed in 1% osmium tetraoxide for 1 h. The samples were examined with a transmission electron microscope (JEM-1200EX, JEOL Co., Japan).

***Cell Culture***

HepG_2_ cells were maintained in MEM (Gibco) supplemented with 10% FBS (Gibco). Hepatocytes were isolated from mice using the two-step collagenase perfusion protocol [2]. The cells were treated with TSH for 24 h. transfected with the pcNDA 3.1-AK044604 overexpression plasmids and vector control (Shanghai Integrated Biotech Solutions Co., Ltd.) using the Lipofectamine^®^ 3000 Transfection Kit (Invitrogen). The medium was changed 8-12 h after transfection according to the manufacturer's directions. The cells were treated with plasmids or SRT1720 in different experiments for 8 h, and then treated with TSH for another 24 h. TSH treatment was given after the medium was changed in transfection experiment. TSH (T8931-1VL) and SRT1720 were purchased from Sigma and MCE.

**In Situ *Detection of Mitochondrial ROS***

We dropped MitoSox Red (1:2000, Invitrogen) on cryostat sections and cells as described by Du [1] with modifications. The sections or cells were washed three times, incubated with the dye at 37 °C for 30 minutes, and then washed three times. The entire process should be fast and gentle. After that, we examined them under a laser confocal microscope or fluorescence microscope immediately after mounting. Images were analyzed and fluorescence intensity was quantified using ImageJ software as described by the researchers [4-6].

***Real-Time PCR***

Total RNA was extracted from mouse livers and cells using TRIzol (TaKaRa) and subjected to reverse transcription kits (TaKaRa). The resultant cDNA was used as a template for real-time quantitative PCR conducted with the Light Cycler system (Roche). 18s RNA or β-actin was utilized as an internal control for lncRNA and SIRTs respectively for normalization. The oligonucleotide primers used were shown in Supplementary Table S1.

***Western Blot Analysis***

For whole protein extraction, liver homogenates and cells were lysed in lysis buffer containing a protease inhibitor. Furthermore, liver homogenates and cells were fractionated into mitochondrial and cytoplasmic fractions as described in the text. Detection was performed using Fluor ChemQ (Cell Biosciences). Densitometric analysis of the scanned blots was measured using ImageJ software and the results were expressed as fold change relative to the control after normalization to their respective internal control. Antibodies against CypD, SIRT3 and COX IV were obtained from Abcam. Acetylation antibodies and antibodies against cytochrome c, SIRT1 and β-actin were purchased from Protein Tech.


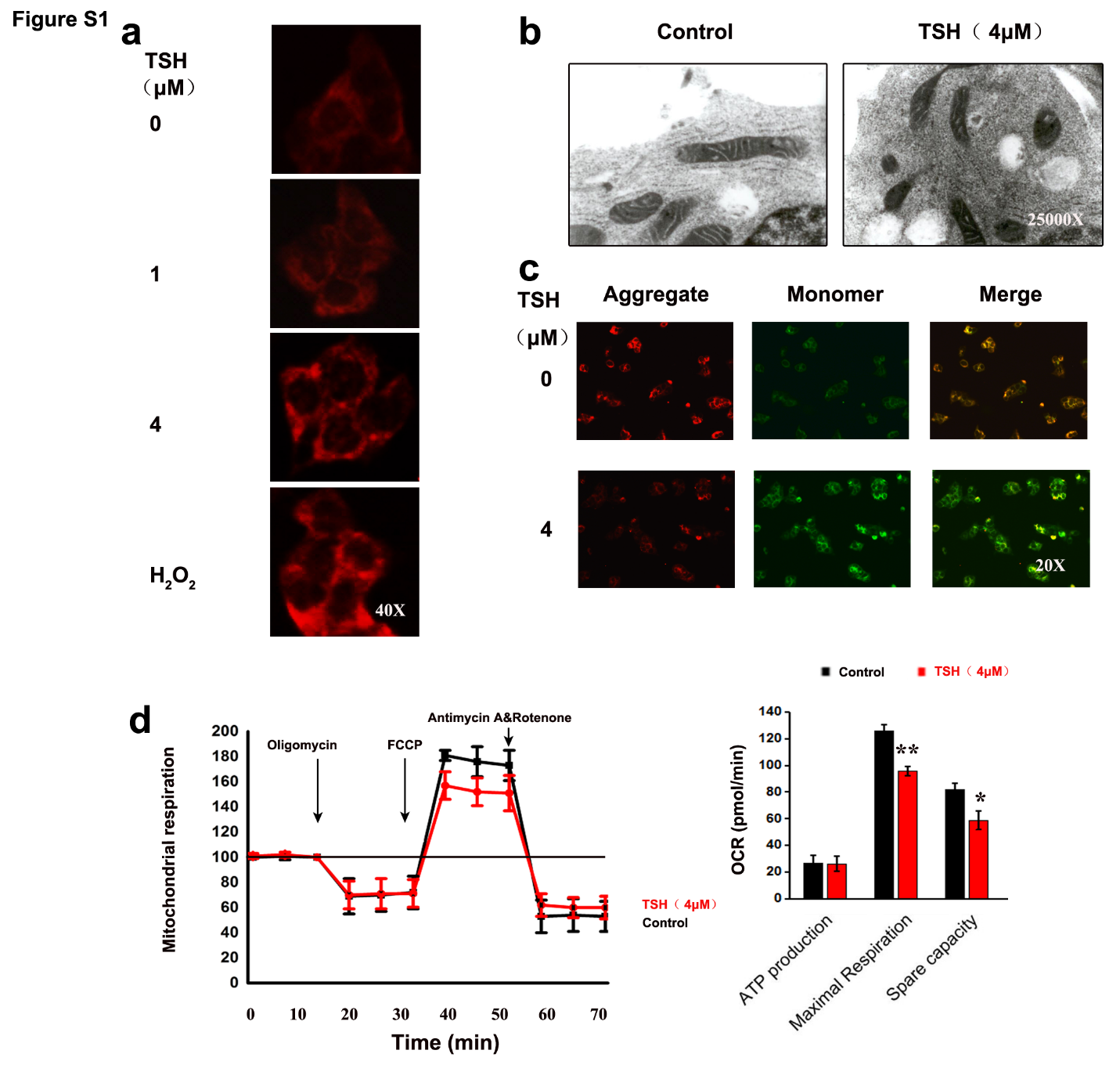


Figure S1: TSH triggers mitochondrial stress in HepG_2_ cells. HepG2 cells were cultured in pretreatment medium with or without TSH. (a) Representative images of MitoSox Red staining in HepG_2_ cells by immunofluorescence microscopy. H_2_O_2_ was used as a positive control. (b) Representative images of TEM showing impairment of mitochondria in TSH-treated cells. (c) JC-1 staining.

(d) Cells were seeded in XF Cell Culture Microplates. Mito Stress Test parameters from Wave data were exported to Excel, and ATP production, the maximal respiration, and spare respiratory capacity were analyzed. The data are presented as the mean±SD. **p<*0.05, ***p<*0.01 versus the control group.


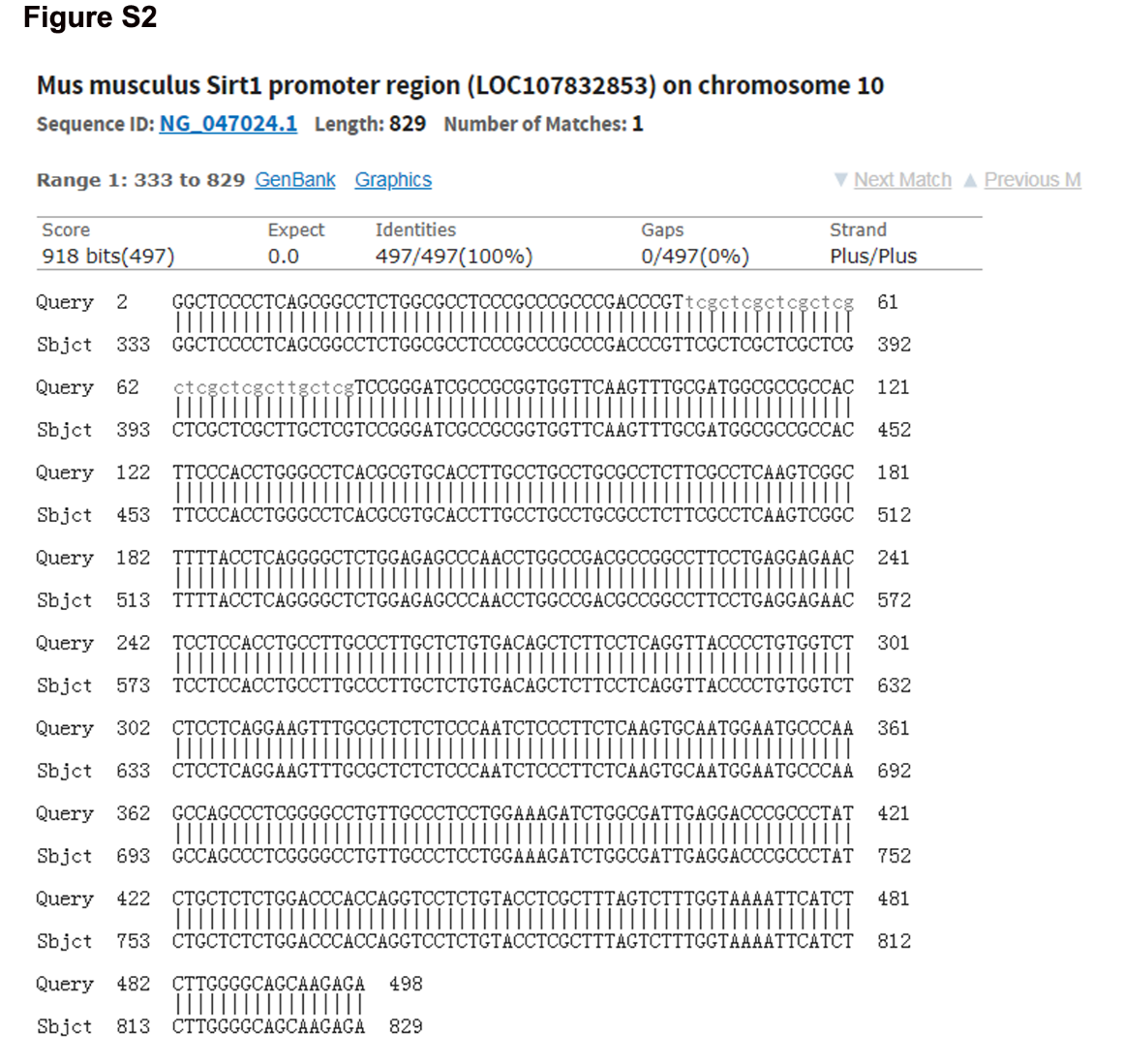


Figure S2: NCBI’s BLAST alignment showed that a segment of 497 nucleotides was found 100% identical in AK044604 and the promoter region of SIRT1.


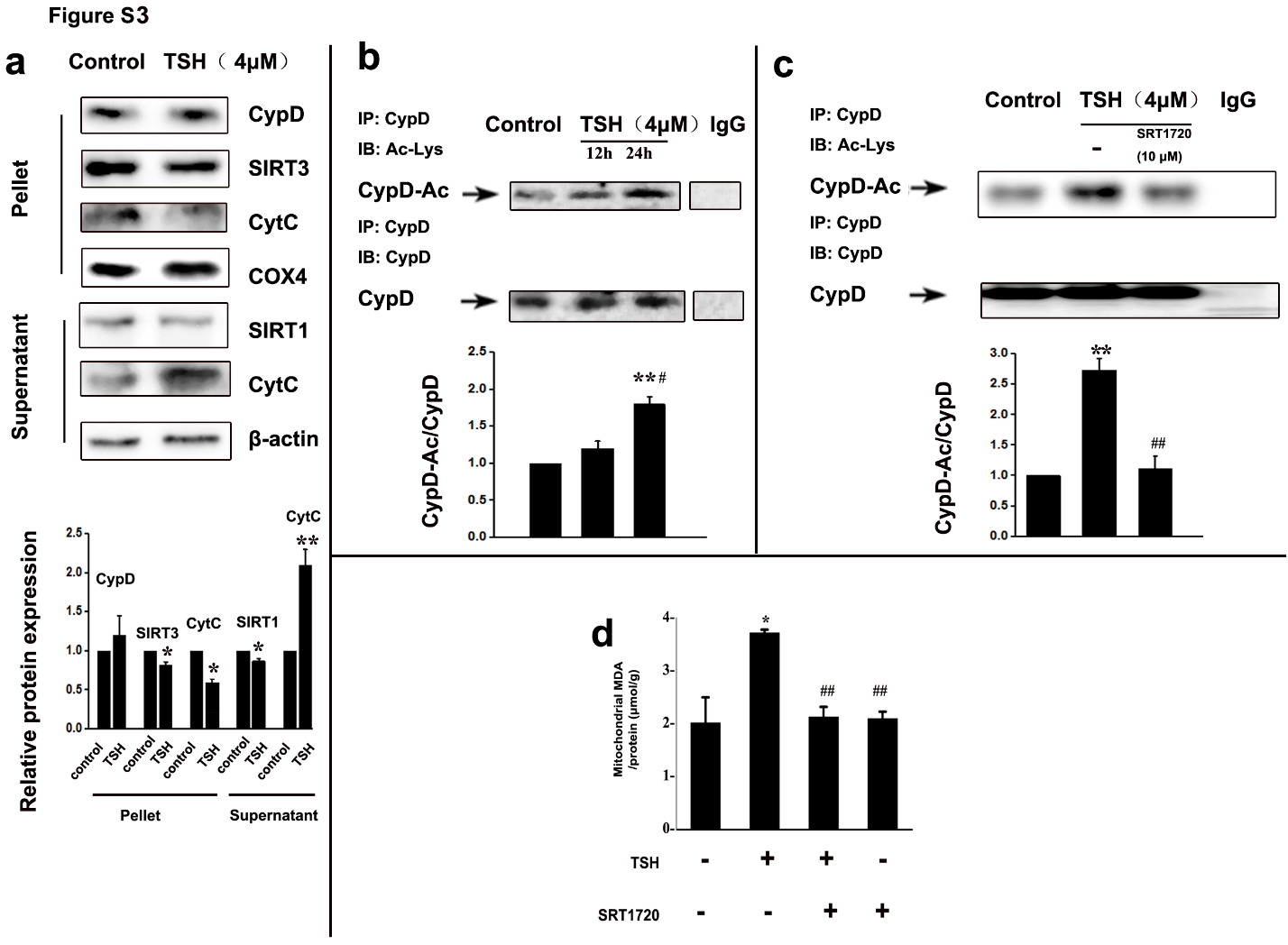


Figure S3: SIRT1/SIRT3/CypD signaling is involved in TSH-induced mitochondrial stress *in vitro*. (a) Representative images of Immunoblotting (upper) and quantification (lower) in the mitochondrial (pellet) and cytosolic (supernatant) fractions of TSH-induced mouse primary hepatocytes. (b-d) HepG_2_ cells. (b-c) Representative images of Immunoblotting (upper) and quantification (lower) for CypD acetylation. (d) MDA levels. The data are presented as the mean±SD. **p<*0.05, ***p<*0.01 versus the control group; #*p<*0.05, ##*p<*0.01 vs the TSH group.

Table S1. The oligonucleotide primer list.

| Gene |  | Forward primer | Reverse primer |
| --- | --- | --- | --- |
| Mouse |  |  |  |
| AK044604 |  | TGTGACTTGGGCGATTGACT | TCCCTCTCTCTAGGGCTTCG |
| SIRT1 |  | ATCTTCACCACAAATACTGCCAAG | CTCCATAATACAAGGCTAACACC |
| SIRT3  18s RNA |  | GCTGCTTCTGCGGCTCTATAC  AGGGGAGAGCGGGTAAGAGA | GAAGGACCTTCGACAGACCGT  GGACAGGACTAGGCGGAACA |
| *β*-actin |  | GGCTGTATTCCCCTCCATCG | CCAGTTGGTAACAATGCCATGT |

**References**

[1] H. Du, L. Guo, F. Fang *et al*., "Cyclophilin D deficiency attenuates mitochondrial and neuronal perturbation and ameliorates learning and memory in Alzheimer's disease," *Nature Medicine,* vol. 14, no. 10, pp. 1097–1105, 2008.

[2] X. Wang, H. Du, S. Shao *et al*., "Cyclophilin D deficiency attenuates mitochondrial perturbation and ameliorates hepatic steatosis," *Hepatology,* vol. 68, no. 1, pp. 62–77, 2018.

[3] F. Yan, Q. Wang, M. Lu *et al*., "Thyrotropin increases hepatic triglyceride content through upregulation of SREBP-1c activity," *Journal of Hepatology,* vol. 61, no. 6, pp. 1358–1364, 2014.

[4] W. Qin, M. Luo, K. Wang, *et al.*,"A combined treatment with erythrocyte lysis solution and Sudan Black B reduces tissue autofluorescence in double-labeling immunofluorescence," *Microscopy*, pp. 1–11, 2018.

[5] X. Sun, Y. Mao, P. Dai, *et al.*, "Mitochondrial dysfunction is involved in the aggravation of periodontitis by diabetes," *J Clin Periodontol*, vol. 44, no. 5, pp. 463-471, 2017.

[6] M. Melcher, K. Danhauser, A. Seibt, *et al.*," Modulation of oxidative phosphorylation and redox homeostasis in mitochondrial NDUFS4 deficiency via mesenchymal stem cells,” *Stem Cell Research & Therapy,* vol. 8, pp. 150, 2017.
